# Green and Sustainable Manufacture of Ultrapure Engineered Nanomaterials

**DOI:** 10.3390/nano10030466

**Published:** 2020-03-05

**Authors:** David Ortiz de Zárate, Carlos García-Meca, Elena Pinilla-Cienfuegos, José A. Ayúcar, Amadeu Griol, Laurent Bellières, Esther Hontañón, Frank E. Kruis, Javier Martí

**Affiliations:** 1 Valencia Nanophotonics Technology Center, Universitat Politècnica de València, 46022 València, Spain; cargarm2@ntc.upv.es (C.G.-M.); epinilla@ntc.upv.es (E.P.-C.); jayucar@ntc.upv.es (J.A.A.); agriol@upvnet.upv.es (A.G.); blaurent@ntc.upv.es (L.B.); jmarti@ntc.upv.es (J.M.); 2 Grupo de Nanosensores y Sistemas Inteligentes (NoySI), CSIC, 28006 Madrid, Spain; esther.hontanon@csic.es; 3 Institute of Nanostructures and Technology, University Duisburg-Essen, 47057 Duisburg, Germany; einar.kruis@uni-due.de

**Keywords:** green, engineering, nanomaterials, tunable, nanoclusters, nanoalloys, nanostructures

## Abstract

Nanomaterials with very specific features (purity, colloidal stability, composition, size, shape, location…) are commonly requested by cutting-edge technologic applications, and hence a sustainable process for the mass-production of tunable/engineered nanomaterials would be desirable. Despite this, tuning nano-scale features when scaling-up the production of nanoparticles/nanomaterials has been considered the main technological barrier for the development of nanotechnology. Aimed at overcoming these challenging frontier, a new gas-phase reactor design providing a shorter residence time, and thus a faster quenching of nanoclusters growth, is proposed for the green, sustainable, versatile, cost-effective, and scalable manufacture of ultrapure engineered nanomaterials (ranging from nanoclusters and nanoalloys to engineered nanostructures) with a tunable degree of agglomeration, composition, size, shape, and location. This method enables: (1) more homogeneous, non-agglomerated ultrapure Au-Ag nanoalloys under 10 nm; (2) 3-nm non-agglomerated ultrapure Au nanoclusters with lower gas flow rates; (3) shape-controlled Ag NPs; and (4) stable Au and Ag engineered nanostructures: nanodisks, nanocrosses, and 3D nanopillars. In conclusion, this new approach paves the way for the green and sustainable mass-production of ultrapure engineered nanomaterials.

## 1. Introduction

Today, materials are scaled down to the nanometer scale in the search for exciting new properties arising from confinement effects and the change in surface to volume ratio [1], reaching a global market of 11 million tonnes and €20 billion, according to the European Commission. Frequently, the desired properties critically rely on a precise control over material’s nanoscale features such as the degree of agglomeration, composition, size, shape and assembly/location [2,3,4,5,6]. Therefore, tunable or engineered nanomaterials have become highly appreciated building blocks for cutting-edge technologic applications [2], and their green and sustainable mass production according to nanomanufacturing criteria—scalability, reliability and commercial viability—[7], is a matter of environmental, economic, industrial, and societal concern, which is furthermore supported by the European Commission, strongly committed to finance “the green transition”. Small nanomaterials (nanoclusters, NCs, nanoalloys…) might be smartly combined to engineer nanoparticles (NPs) or nanostructures with fine tunability of their chemical and physical properties [8]. However, according to experienced researchers [3] and European roadmaps on nanoparticles [2] and nanomaterials [6], one of the main challenging technological/engineering barriers for the development of nanotechnology is preserving an accurate control over the nanoscale features when scaling-up the production of nanomaterials.

Top-down lithographic processes represent interesting solutions to achieve sophisticated nanostructures which, however, imply the use of expensive equipment and clean rooms, as well as a considerable material waste (downscaling the object size to the nanoscale). On the contrary, bottom-up approaches are cheaper, imply less/no material waste and also allow control at the nanoscale. However, bottom-up routes (in the liquid or the gas phase) exhibit several limitations when scaling up the production [3]. The main problem of gas-phase processes is the agglomeration of NPs, as it is promoted under the conditions leading to a high production rate. Besides using solvents, solution-based syntheses (wet routes) try to reduce the degree of agglomeration by means of surface-active compounds, thus surfactant and solvent molecules remain to a certain extent on the surface of products. As a consequence, wet routes lead to less pure nanomaterials than gas-phase approaches, and require further purification by means of batch processes, becoming not so green, continuous, cost-effective, nor scalable methods [3]. In addition, solution-based approaches often require a “synthesis-then-positioning” strategy to manufacture and position engineered nanostructures, ENSs, involving tip-based expensive equipment [9]. Greener methods in between them, based on the use of supercritical fluids, allow high purity nanomaterials with more environmentally benign solvents such as supercritical CO_2_, water, or ethanol, although they demand expensive high-pressure equipment and more processing steps and reagents [10]. All those limitations are addressed within this study by means of a greener, sustainable, industrially scalable, versatile and cost-effective dry route able to manufacture tunable non-agglomerated nanoclusters, nanoalloys and ENSs in the gas phase, overcoming the main barrier for the development of nanotechnology.

Among the bottom-up approaches towards NP aerosols in the gas phase [2,3,4,5,6], atmospheric pressure spark discharge is chosen due to its low cost and high simplicity, purity, robustness, environmental friendliness and efficiency. While gas-phase atmospheric spark discharge generators (SDGs) are widely used to obtain agglomerated NPs [11,12,13,14,15,16,17], smart strategies have been designed for the fabrication of small non-agglomerated NPs, but they neither meet manufacturability requirements, nor allow control over some nanoscale features.

**Figure 1:**
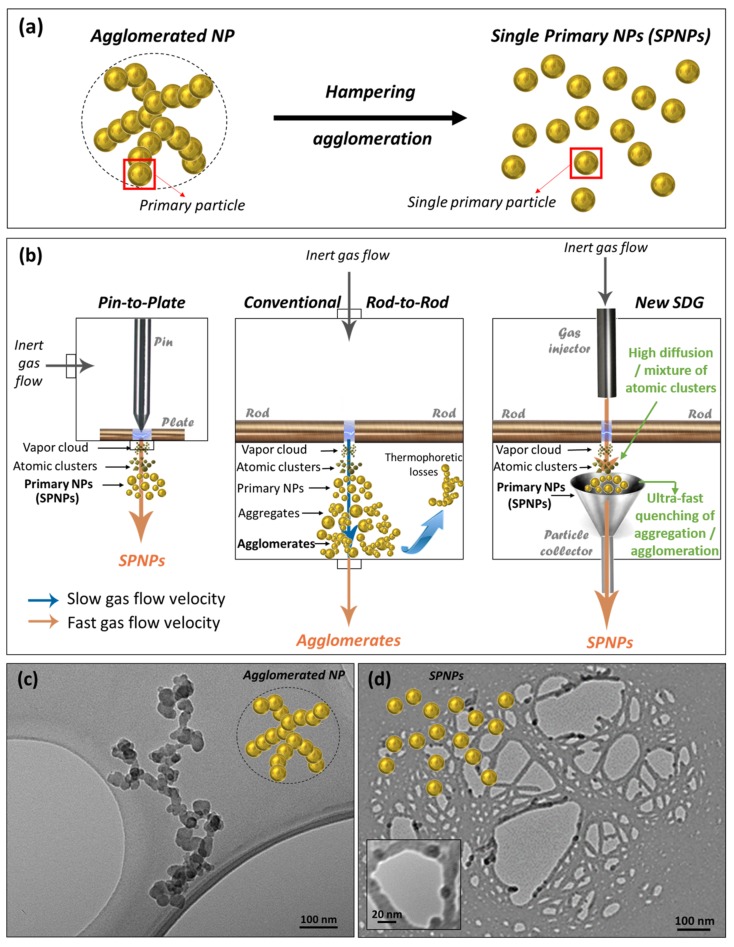
(**a**) Single primary NPs production by hampering agglomeration of primary particles. (**b**) Effect of different SDG geometries on the gas flow velocity and NP transport. (**c**,**d**) TEM images of: (**c**) agglomerated Ag NPs produced with a conventional rod-to-rod SDG, and (**d**) non-agglomerated Ag SPNPs manufactured with the proposed rod-to-rod SDG.

Conventional rod-to-rod SDGs exploit the coagulation and coalescence of agglomerated primary NPs produced by spark to obtain compact spherical NPs [15], which nevertheless require a differential mobility analyzer (DMA) for NP size-selection (implying NP waste) followed by a high temperature furnace for NP sintering. Further, only composition and size are controlled to a certain extent. A single-step process avoiding those requirements, such as quenching the growth of primary NPs to get non-agglomerated nanoclusters (or single primary NPs, SPNPs, Figure 1a), as soon as they form in the plasma, would lean towards green nanomanufacturing criteria. 

This is difficult for conventional rod-to-rod SDGs [13], whose reactor geometry (limiting the carrier gas velocity from the spark region to the chamber exit) and gas flow rates (a few standard liters per minute, slm) induce long residence times, promoting NP aggregation, agglomeration and losses to the walls (by diffusion and thermophoresis) [18,19]. There have been some attempts to drastically reduce the residence time and agglomeration rate (by a factor of 100) [20], by diluting the aerosol downstream of the plasma region with a cold gas flow (by a factor of 10). This approach, however, leads to high gas waste, a large pressure drop in the system, and does not achieve the same level of tunability.

Other configurations such as pin-to-plate, wire-in-hole and rod-to-tube SDGs [18,19,21], overcome part of these limitations, as they benefit from a reactor geometry that allows a higher local carrier gas velocity where NPs form, which reduces the agglomeration rate and the residence time, thus quenching NP growth. Nevertheless, these processes are difficult to transfer to the industrial scale, since pins, wires, sharpened rods, and plates wear with time, thus changing nano-scale features and demanding periodic replacement, both hampering continuous stable NP production. On the contrary, the rod-to-rod configuration exhibits higher industrial scalability, production rates (from 0.1 to 69 g/h), and automation capacity [16,22,23] than using long electrode rods and a step motor to keep the interelectrode distance constant, enabling continuous stable NP production over longer times.

Here, a new design shortening the reactor residence time and gathering all the desired features is proposed, enabling greater nanomanufacturability and tunability within a greener procedure. Figure 1b provides a comprehensive comparison of pin-to-plate, rod-to-rod and the new SDG reactor chambers, from a geometric point of view. First, the proposed SDG keeps the rod-to-rod configuration to ensure the long-term continuous stable production of NPs and other nanomaterials. Besides, the new SDG includes a particle collector at the spark area, allowing a higher local carrier gas velocity where NPs form (depicted by means of a red arrow), enabling a faster transport of the newborn SPNPs out of the reactor. This induces a drastic dilution and abatement of their temperature and residence time (minimizing NC collisions thus quenching their coagulation and coalescence) in a sustainable way, promoting several advantages: (1) less agglomeration (allowing the obtention of SPNPs), (2) less aggregation (smaller NCs), (3) less thermophoretic losses, (4) better mixture of atomic clusters, driving to more homogeneous nanoalloys, and (5) higher control over NCs availability during growth process (opening doors to shape control in the gas phase). Thus, the new SDG not only avoids NP coagulation and coalescence, but also allows tuning nanoscale features such as composition, size, shape and location to an unprecedented extent within the gas phase, by means of a greener and sustainable process which is more compatible with mass-production. As a proof of the critical influence of the reactor geometry on the agglomeration rate, Figure 1c shows agglomerated Ag NPs obtained with a conventional rod-to-rod SDG, collected on a transmission electron microscopy (TEM) grid. Remarkably, non-agglomerated, smaller Ag SPNPs were obtained under the same conditions (described in the following section) using the new SDG (Figure 1d). 

## 2. Materials and Methods 

### 2.1. SPNPs Nanomanufacture

**Figure 2:**
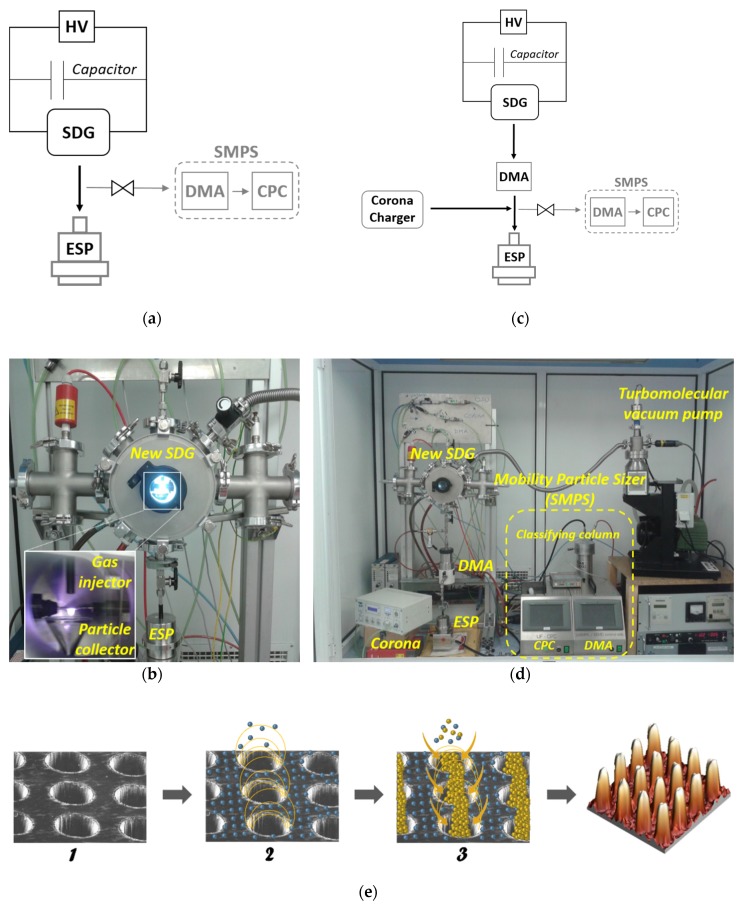
(**a**) Scheme and (**b**) experimental setup for SPNPs and non-agglomerated NPs manufacture. (**c**) Scheme and (**d**) experimental setup for Experimental setup for ENSs production, including the particle measurement system. (**e**) Scheme for a nanoparticulated 3D assembly through the new SDG.

A particle collector made of glass (or metal-sputtered glass) is placed under the plasma zone of a rod-to-rod SDG reactor in horizontal cross-flow configuration with a stainless steel ¼ inch gas injector 5 mm over the plasma zone, in order to reduce the reactor residence time, providing the newborn SPNPs with a fast transport out of the reaction chamber (Figure 2a,b). The manufacture of SPNPs was conducted using metal electrodes of high purity (99.99% 3 mm Au rods, 4 and 10 mm Ag rods, all of them of 30 mm length, ChemPur GmbH, Karlsruhe, Germany), separated 1–2 mm from each other. The electrodes were connected to a RCL circuit (C = 15 nF) and a high voltage power supply (DC: CCR5-P-300 (Technix, Créteil, France), 0–5 kV, 0–120 mA, or a low-cost AC: 0-096-600-017 (Beru, Ludwigsburg, Germany), 15 kV, 40 mA). A continuous gas flow (ranging from 1 to 6 standard liters per minute, slm) was passed through the interelectrode gap to generate the plasma (when the breakdown voltage is reached), to dilute and cool the SPNPs down, and also to drive them out of the system as fast as possible (quenching their growth). When SPNPs of a particular size were required, a DMA was employed to select a narrower size distribution interval. The aerosol was monitored by means of a scanning mobility particle sizer (Figure 2d, U-SMPS 2000, Palas GmbH, Karlsruhe, Germany, counting on a long classifying column, a DMA, and a condensation particle counter, UF-CPC 100), and the experiments were conducted with particle concentrations of 10^7^ particles/cm^3^. The synthesis of agglomerated NPs was conducted by removing the particle collector under the same conditions.

### 2.2. Non-Agglomerated NPs Nanomanufacture

The new rod-to-rod SDG reactor was directly connected to an electrostatic precipitator (ESP, Figure 2a,b), in order to deposit the quenched newborn SPNPs on different substrates (Si, SiO_2_, Al foil, ITO/SiO_2_ glass and TEM grids). SPNPs were also deposited on substrates placed under the spark region (over the particle collector), inside the SDG chamber.

### 2.3. ENSs Nanomanufacture

The precise location of NPs requires a charge distribution pattern on the substrate surface, and cheap techniques such as nanoimprint could be used for this. However, we employed lithographic methods only for demonstration purpose. The substrate (Si, SiO_2_, and ITO/SiO_2_ glass) was covered with a resist (usually PMMA) by means of an EVG101 spin coater (EV Group, St.Florian am Inn, Austria), and the pattern of interest was transferred by means of e-beam lithography. An optimized direct writing electron beam process is applied on a 100 nm thick layer of PMMA 950 K positive resist, using a Raith150 equipment (Raith, Dortmund, Germany) operating at 10 KeV, with aperture size of 30 microns. After exposure, the resist is developed by immersion in a mixture of isopropanol and methyl isobutyl ketone (IPA-MIBK, 50 s), and the reaction is subsequently stopped in IPA (30 s). All reagents were purchased from Panreac (Castellar del Vallès, Spain). Once the pattern is transferred (Figure 2e1), negative air ions from an aerosol corona charger (Ioner CC-8020, Ramem, Torrejón de Ardoz, Spain) are conducted to the substrate, which is placed in an ESP connected to a positive power supply, in order to electrostatically charge the photoresist over time, driving to a negative surface charge of PMMA, while the openings remain at positive potential (Figure 2e2). After that, the modified rod-to-rod SDG is connected to a DMA to select negatively charged SPNPs, which are driven to the ESP, still connected to a positive power supply (Figure 2c). Electrostatic forces (the negative charge of the PMMA surface and the positive potential in the openings) focus negative SPNPs to the pattern openings, acting as nanoscopic lenses, filling the pattern of interest with SPNPs (Figure 2e3).

### 2.4. Characterization

SEM images were attained with a S-4500 Field Emission microscope (Hitachi, Chiyoda, Tokyo, Japan). TEM images are acquired with a JEM-1010 microscope (JEOL Ltd., Akishima, Tokyo, Japan) operated at 100 kV, while HRTEM, HRSTEM, and EDX elemental mapping images were obtained with a JEM-2100 F microscope (JEOL Ltd., Akishima, Tokyo, Japan) using an accelerating voltage of 200 kV. AFM images are attained with a multimode Dimension 3100 microscope (Veeco Digital Instruments/Bruker, Billerica, MA, USA), operated in tapping mode with Silicon tips from Nanosensors, with force constant k of 42 N/m, and a resonance frequency of 300 kHz, and then processed with WSxM software (WSxM 5.0 develop 8.0 2015, Nanotech Electrónica S.L., Spain) [24]. 

## 3. Results and Discussion

**Figure 3:**
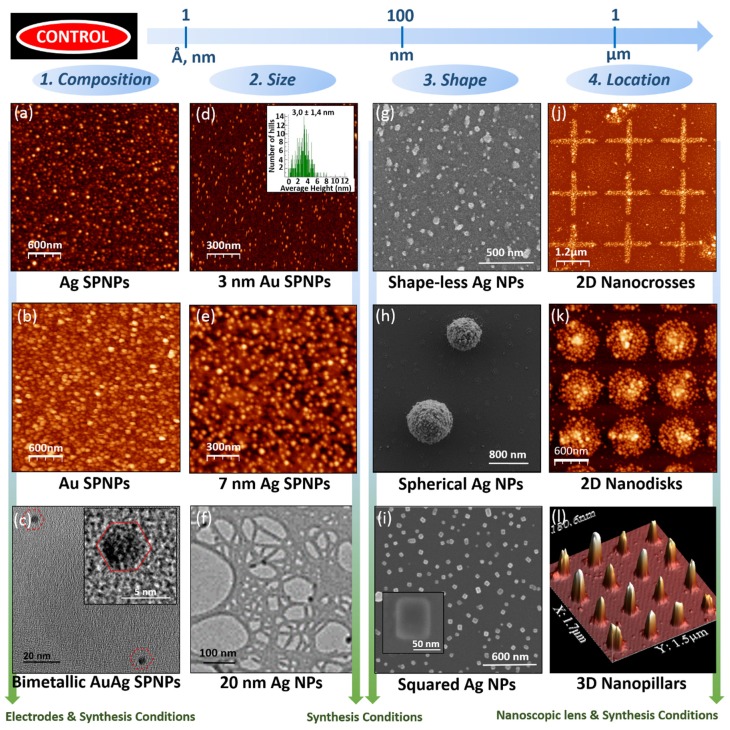
Tuned composition, size, shape and location of SPNPs, NPs and 3D ENSs manufactured by means of the modified SDG. The most suitable imaging technique is used in each case: AFM (**a**,**b**,**d**,**e**,**j**–**l**), TEM (**c**,**f**) and SEM (**g**–**i**).

Thus, experimental demonstration of the high degree of tunability gained at five different levels (Figure 1d and Figure 3), while preserving a green nanomanufacturability is provided, opening doors to the sustainable mass-production of many different ultrapure engineered nanomaterials.

### 3.1. Engineering Composition

Metal NPs display different physicochemical properties depending on their nature. Consequently, bimetallic NPs are of particular interest, as they may offer intermediate or synergistic features, leading to the modulation of their properties (as requested, e.g., in photonics and plasmonics) or even to the enhancement of their activity and selectivity (as demanded in catalysis) [25,26].

Metal nanoalloys have been previously obtained by means of electrical discharges (from alloy electrodes or employing two electrodes of different metals) by different strategies [27]. However, these mainly lead to agglomerated NPs [28,29,30,31], and require additional sintering/crystallization steps [15] to finally obtain non-agglomerated small bimetallic NPs (not SPNPs anymore). Some authors describe internally mixed NPs, using a conventional rod-to-rod SDG with electrodes of different composition [32]. Nevertheless, this gives rise to a broad NP composition distribution, which is explained in terms of the oscillatory behavior of discharges, inducing electrodes to take turns to emit vapor clouds. This leads to single-component particles of 3 nm from each vapor cloud, which subsequently coagulate with other single-component particles (of different or the same metal), promoting bigger, internally mixed NPs with variable composition distribution.

Unlike the aforementioned procedures, the new method leads to a faster vapor diffusion in the vicinity of the spark by using the proposed SDG, allowing a better and earlier mixture of metal NCs, reducing segregation, agglomeration rate, and residence time, while quenching the growth of primary NCs with lower gas flow rate. 

As a demonstration, ultrapure Au, Ag, and bimetallic AuAg SPNPs were prepared. Figure 3a,b displays ultrapure Ag and Au SPNPs under 10 nm (obtained with cathode and anode electrodes of the same material), electroprecipitated on a silicon wafer. Bimetallic SPNPs were produced with Au and Ag electrodes, and then deposited them on TEM grids, in order to characterize them by high resolution TEM (HRTEM) and high-resolution scanning TEM (HRSTEM).

**Figure 4:**
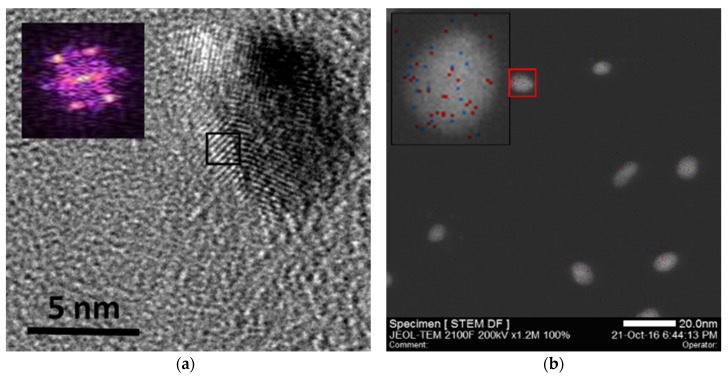

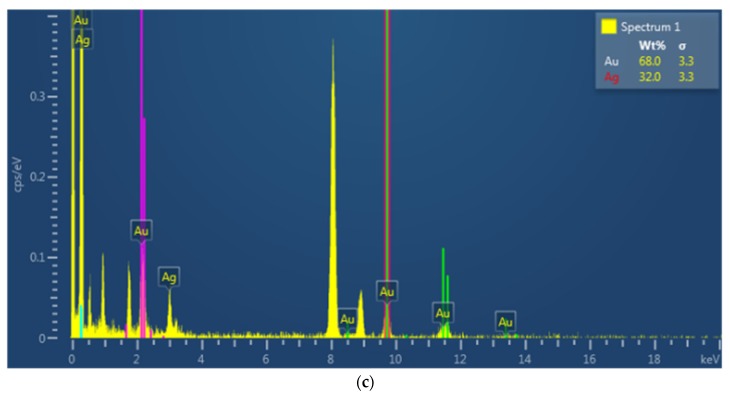
(**a**) HRTEM and (**b**) HRSTEM images of bimetallic AuAg SPNPs. Inset in Figure 4a displays the FFT pattern of the crystal lattice inside the black square. Inset in Figure 4b shows the EDX elemental mapping of the bimetallic SPNP inside the red square. (**c**) EDX elemental analysis of the bimetallic SPNP inside the red square.

Figure 3c presents two 5-nm AuAg SPNPs exhibiting a face centered cubic (fcc) arrangement of atoms. This was confirmed by studying their crystal lattice shown in Figure 4a, and the corresponding fast Fourier transform (FFT) pattern displayed in the inset. Energy-dispersive X-ray spectroscopy (EDX) elemental mapping performed in HRSTEM (Figure 4b) and the corresponding elemental analysis (Figure 4c) of the nanoparticles confirmed not only the bimetallic nature of these SPNPs (with compositions around 68% of Au and 32% of Ag), but also their homogeneous composition distribution, with an average composition distribution of %Ag/%Au = 0.48 and a standard deviation of 0.02, which is an order of magnitude lower than previous results obtained by means of a gas-phase process without pre-alloyed sources [32].

Thus, the proposed SDG allows for tuning the composition of SPNPs, from non-agglomerated ultrapure monometallic to bimetallic nanoparticles, providing a greener route to multi-metallic nanoparticles able to explore other compositions, such as ceramic or even composite materials, by a careful choice of the electrodes materials (not restricted to alloy ones), atmosphere, and production parameters, in order to obtain specific non-agglomerated homogeneous nanoalloys on demand.

### 3.2. Engineering Size

SPNPs and non-agglomerated NPs of different sizes, ranging from very few to tenths of nm, were manufactured. First, Au NCs with an average particle size as low as 3 nm (Figure 3d) were produced with gas flow rates of only 5 slm, while conventional rod-to-rod SDGs require 33 slm [20]. The ultrafast gas flow velocity provided by the modified SDG in the spark region promotes a quicker dilution, reducing the number of primary NCs collisions to a very small extent, quenching aggregation, agglomeration and the size of SPNPs with the lowest required flow rate of inert gas, minimizing cost and waste (greener approach). Furthermore, bigger NPs (7–20 nm) were manufactured by promoting primary nanocluster collisions, employing lower gas flow rates and higher power (Figure 3e,f), and even bigger NPs are presented in the following section by promoting subsequent growth. Therefore, the new SDG together with careful control over the production parameters make the size of non-agglomerated NPs tunable, from NCs to NPs, in a green and sustainable manner.

### 3.3. Engineering Shape

Control over NPs shape was also demonstrated, enabling the manufacture of non-agglomerated NPs with amorphous, spherical or squared shape by a green gas-phase process, without additional sintering steps. 

Surface energy minimization principle governs the growth of very small NCs such as SPNPs, which can be understood as primary NCs made of metal atoms arranged in fcc structures, whose growth has been quenched when they were acquiring close-packed 3D polyhedron geometries with low surface-to-volume ratio. Once NPs reach a certain size, the proportion of surface atoms becomes so small that the lattice energy dominates. As a consequence, if those SPNPs undergo a subsequent growth/assembly (i.e., when deposited on a substrate for a certain time), metastable high surface-to-volume structures can be obtained. To this end, the surface reactivity as well as the monomer diffusion to the surface must be carefully controlled [33]. 

On one hand, the concept of surface reactivity deals with specific chemi or physisorption of molecules on certain cluster facets, which effectively reduce the specific surface free energy, altering the ratios between the surface free energy of different facets. As a consequence, physisorbed gases create poisoned facets that will not be reactive, while more energetic facets are more prone to react, allowing cluster growth in preferential directions. As examples of this, cuboctahedron nanoparticles (nearly spheres), truncated cuboctahedrons (close to cubes), and faceted particles of fcc crystals have been grown in nitrogen, hydrogen or argon environment respectively [34,35,36,37].

On the other hand, the concept of monomer diffusion to the surface refers to a rearrangement of material from energetic facets of the cluster to lower energy positions, in order to minimize the surface energy [33]. According to nucleation and growth theory, when a low deposition rate of monomers is applied (SPNPs in this case), there is time for diffusion to take place, allowing a reaction-limited growth mechanism which leads to compact geometries following surface energy minimization criteria imposed by the different facets of the growing clusters. On the contrary, a high deposition rate of monomers enables a diffusion-limited growth mechanism producing opened structures with fractal geometries.

Therefore, the nature of the inert gas (enabling or disabling cluster growth in preferential directions) [34,35,36,37] and the SPNP deposition rate (promoting reaction-limited or diffusion-limited growth) [33] were the key parameters chosen to control the surface reactivity as well as the monomer diffusion to the surface, respectively. 

Following these criteria, growth conditions enhancing isotropy were selected in order to produce low surface-to-volume spherical NPs: nitrogen (displaying no facet preferentiality in fcc clusters) was chosen as the inert gas, and a moderated SPNPs deposition rate was used (applying 10 mA and 0.3 kV during SPNPs production), enabling the diffusion of material to the cluster’s surface. This allowed a reaction-limited growth mechanism, inducing the formation of 3D close-packed spherical Ag NPs (Figure 3h). 

With the aim of promoting anisotropy, nitrogen was substituted by argon as the inert gas (with facet preferentiality in fcc clusters). Then, a high concentration of Ag atoms (using 10 mA and 2.5 kV) was employed, enabling the production of a great amount of complex 3D clusters with different arrangements. This favored a diffusion-limited mechanism of growth that induced the fast and irreversible agglomeration of SPNPs, and thus the formation of shape-less Ag NPs (Figure 3g). 

Finally, when a low concentration of Ag atoms was employed under an argon environment (using low current, 1 mA, and low voltage, 0.3 kV) to produce and deposit SPNPs, there was enough time for the diffusion of material to less energetic facets. Thus metastable high surface-to-volume structures with lower lattice energy such as Ag single nanocubes (Figure 3i) were obtained, demonstrating NP shape control by means of a green gas-phase process such as SDG without additional sintering steps.

### 3.4. Engineering Location

Control over the location of tunable SPNPs was demonstrated by focusing them through nanoscopic lenses achieved by the IAAL strategy [38,39] thanks to their synthesis in the gas phase, in order to build up ultrapure well defined stable 3D ENSs (although simpler strategies to focus the SPNPs could be used, among all those described in the bibliography) [40,41,42,43,44,45]. As a consequence, NCs manufactured by the new SDG were employed as the greenest and smallest tunable building blocks able to maximize the resolution of 3D ENSs and precisely control their physicochemical properties. Negatively charged SPNPs selected by a DMA were focused to the silicon openings on a substrate with a patterned layer of negatively charged resist, by electrostatic forces. Following this procedure, very stable 2D nanostructures such as nanoparticulated Ag nanocrosses (Figure 3j) and nanodisks (Figure 3k) were obtained. Upon further deposition of SPNPs, complex, very stable 3D ENSs were also achieved, such as Au nanopillars (Figure 3l). Note that some applications require a subsequent lift-off process. In this sense, it is worth mentioning that these 3D ENSs persisted after this aggressive treatment without any further process such as metallization, frequently employed as protection or enhancer for SERS or photonic sensing applications [39].

## 4. Conclusions

In summary, the novel reactor design overcomes the main problem of gas-phase nanomaterials reactors (agglomeration) as well as the main technological barrier for the development of nanotechnology (tuning nano-scale features when scaling-up the production of nanoparticles/nanomaterials) in a green and sustainable manner. To do so, the new SDG counts on a shorter residence time, addressing the ultra-fast quenching of primary particles growth, enabling the green and sustainable nanomanufacture of an aerosol of small non-agglomerated NCs or SPNPs, providing several improvements:

➢ Higher sustainability
  A greener and more sustainable process to manufacture engineered nanomaterials (using no solvents, a lower gas flow rate, and reducing material waste)
➢ Higher nanomanufacturability—scalability, reliability and commercial viability
  - Industrially scalable process to manufacture engineered nanomaterials (gathering the best features ensuring a stable continuous production of tunable NPs)
  - Low-cost process to manufacture engineered nanomaterials (avoiding expensive top-down or high-pressure equipment, raising affordability and guaranteeing commercial viability)
  - Versatile process to manufacture engineered nanomaterials (from non-agglomerated NCs and nanoalloys, to 3D ENS)
  - Reduced thermophoretic losses
➢ Higher tunability of manufactured nanomaterials
  - Control over agglomeration (enabling synthesis conditions with lower agglomeration, driving to the sustainable manufacture of SPNPs)
  - Control over aggregation (allowing the production of smaller NCs)
  - Control over the diffusion and mixture of atomic clusters while SPNPs are being formed, enabling a fine tuning of composition and/or improving compositional homogeneity
  - Control over NCs availability during growth process (opening doors to shape control in the gas phase)
  - Tunability over five nanoscale features of nanomaterials: degree of agglomeration, composition, size, shape, and location

These advantages allowed the manufacture of ultrapure:

(1) Single-metal (Ag, Au) SPNPs as well as multi-metal (AuAg) nanoalloys with the narrowest composition distribution (ten times smaller than the gas-phase state of art) among non-solution approaches without pre-alloyed sources, being extensible to other compositions, including ceramic and composite NCs.
(2) Au and Ag NPs measuring 3–20 nm with the lowest gas flow rate described for rod-to-rod SDGs (six times smaller than the state of art), which can be used as tunable building blocks for many applications, as well as the subsequent production of ENSs.
(3) Amorphous, spherical and squared non-agglomerated NPs from 50 to 800 nm, the first shape-controlled ultrapure NPs obtained by a non-solution room-temperature gas-phase process, to the best of our knowledge.
(4) Au and Ag ENSs, such as nanodisks, nanocrosses, and 3D nanopillars with enhanced stability.

Therefore, the novel reactor design enabled a green non-solution bottom-up gas-phase process to manufacture ultrapure NCs, nanoalloys and 3D ENSs with unprecedented tunability (degree of agglomeration, composition, size, shape, and/or location), and nanomanufacturability, suggesting a paradigm shift towards this green, low-cost, sustainable, reliable, and industrially scalable process to reach the mass-production of ultrapure engineered nanomaterials. 

Future research is currently being oriented towards the application of these ultrapure tunable nanomaterials for the fabrication of tunable plasmonic sensors for biomedicine, reconfigurable optoelectronic devices, advanced multi-metallic nanocatalysts, and specific nanoclusters for computing and energy applications.
